# Unilateral Neurogenic Pruritus Following Intracranial Surgery

**DOI:** 10.7759/cureus.8661

**Published:** 2020-06-17

**Authors:** Ankit Agarwal, Adriana Fernandez Bowman

**Affiliations:** 1 Pediatrics, Ascension Sacred Heart Hospital, Pensacola, USA; 2 Pediatric Medicine, University of Florida College of Medicine, Pensacola, USA

**Keywords:** pruritus, itch, gabapentin, intracranial, surgery

## Abstract

Itch is a frequent complaint reported by the patients and is usually ascribed to dermatological causes. Central neurogenic pruritus remains an under-recognized complication with an unclear etiology previously described in the patients with stroke or with an intramedullary mass of the spinal cord. We describe a case of a nine-year-old male who developed unilateral pruritus seven days after he underwent right hemicraniectomy due to ruptured arteriovenous malformation. The patient manifested a significant improvement in pruritus after starting gabapentin. This report highlights the need for having a high index of suspicion for central neurogenic pruritus in such patients.

## Introduction

Itch is a common symptom and is often associated with dermatological etiologies or medication side effect. Metabolic and endocrinologic disorders are often diagnosed in the absence of dermatological diseases.

In neurological disorders, postherpetic itch after shingles or radiculopathy due to osteoarthritis accounts for most of the part [1,2]. Diseases affecting the central or peripheral nervous system are generally associated with pain and paresthesia rather than pruritus. Central neurogenic pruritus has seldom been reported in the literature with stroke and intramedullary spinal cord masses as the most common etiologies [3]. There is a paucity of information regarding the intracranial surgical procedure as an etiology of central pruritus with no published reports widely available in the literature.

## Case presentation

A nine-year-old male presented with unilateral pruritus seven days after he underwent right hemicraniectomy due to ruptured arteriovenous malformation. The pruritus was unremitting, interfering with daily activities, and refractory to antipruritic medications and emollients. The affected area was localized to the right lower extremity, particularly the L5 dermatome. The patient had no history of renal, liver, endocrine, hematologic, or skin disease. Underlying medical disorders that should be considered in pruritus without a primary cutaneous eruption were excluded by history and physical examination. Laboratory evaluation included complete blood count, liver, and kidney function tests and were all within normal limits. The cutaneous examination was unremarkable. A seizure focus as a cause of paroxysmal pruritus was not identified on an electroencephalogram (EEG). Itching persisted despite discontinuing opioids, which are known to have the side effect of pruritus. The patient was then started on gabapentin tablet 125 mg twice daily. As the patient showed mild improvement in pruritus, the dose was increased to 125 mg three times a day. Two weeks after starting gabapentin, there was a significant improvement in pruritus.

## Discussion

Localized pruritus may be a symptom of any focal neurological phenomenon, such as brain tumors, multiple sclerosis, cerebral vascular aneurysms, and peripheral nerve entrapments. Pruritus tends to be continuous, but may also present in paroxysms or bouts [3]. There is limited information present in the published literature reporting unilateral pruritus after the cranial neurosurgical procedure as in our patient. The excellent initial clinical response to gabapentin supports the diagnosis.

Since central neurogenic pruritus goes unassociated with any established syndrome, and due to its progression to advanced dermatological lesions, the majority of the pertinent case reports have been noticed to be published in dermatology journals. Without diligently giving thought towards the secondary causes, the resulting lesion is mistakenly diagnosed as primary pruritus in such cases.

Although the exact neuroanatomy and neurophysiology of itch perception have not been clarified, it is suggested that itch perception utilizes many of the same neural pathways used in pain sensation [4]. Case reports demonstrate that any type of brain lesion that affects the brain’s itch neurons can cause central neuropathic pruritus, including trigeminal trophic syndrome (TTS) [5]. Further case reports and research may help to understand the exact pathomechanism of this phenomenon.

Treating neurogenic pruritus is a challenge in most of the cases as the treatment effective for conventional itch such as antihistamines and anti-inflammatory is usually ineffective. No high-quality clinical treatment trials have been conducted and no medication has a U.S. Food and Drug Administration indication for neuropathic pruritus [1]. Conservative treatments such as maintaining good skin hydration, cognitive behavioral therapy, and barriers to reduce scratching should be considered in all the patients. Application of topical anesthetics such as lidocaine patches and eutectic mixture of lidocaine and prilocaine (EMLA) cream have been reported to be effective for the peripheral types of neuropathic pruritus [6]. Oral medications are largely untested for neurogenic pruritus. Case reports, mostly in TTS patients, support carbamazepine, gabapentin, amitriptyline, and pimozide [7-9]. Systemic local anesthetics and analogs such as mexiletine are untested but may be considered as they decrease neuronal action potentials, particularly in unmyelinated axons [10].

## Conclusions

Pruritus in patients without clear etiology is a diagnostic and therapeutic challenge for the practitioners. If the dermatological and metabolic tests are unyielding, a detailed history, complete physical examination, and relevant brain and spinal cord imaging are necessary to establish a diagnosis. This report highlights the need for having a high index of suspicion for central neurogenic pruritus in patients with intracranial surgery. In addition, the neurophysiologic basis of pruritus has not been fully established. Further research and reports could provide insights into the ill-defined pathomechanisms of central pruritus which would help to determine better treatment options.
